# Supplementary material for: Multi-objective AGV scheduling in an automatic sorting system of an unmanned (intelligent) warehouse by using two adaptive genetic algorithms and a multi-adaptive genetic algorithm
Source: PLoS One. 2019 Dec 6;14(12):e0226161. doi: 10.1371/journal.pone.0226161 (PMC6897425; doi:10.1371/journal.pone.0226161)
Supplement: S1 Appendix — (PDF) [file pone.0226161.s001.pdf]

```
% Initializing population
function MYinit(population_size, chromosome_size)
global population;
AGV_num=20;
for i=1:population_size
    for j=1:chromosome_size
        if j<=0.5*chromosome_size
            population(i,j) = randperm(AGV_num,1);
        else
            population(i,j) = 60*(0.4+0.1*randperm(11,1));
        end
    end
end
clear i;
clear j;

% Fitness function
function MYfitness(population_size, chromosome_size)
global fitness_value;
global population;
global fitnessSMS_value;
global fitnessNA_value;
global fitnessE_value;

for i=1:population_size
    fitness_value(i) = 0;
    fitnessSMS_value(i) = 0;
    fitnessNA_value(i) = 0;
    fitnessE_value(i) = 0;
    distance1(i)=0;
    distance2(i)=0;
    distance3(i)=0;
    distance4(i)=0;
    distance5(i)=0;
    distance6(i)=0;
    t1(i)=0;
    t2(i)=0;
    t3(i)=0;
    t4(i)=0;
    t5(i)=0;
    t6(i)=0;
end
order=xlsread('order data.xlsx');
order_size=0.5*chromosome_size;
coordinate=zeros(order_size,4);
for i=1:order_size
    a=order(i,1);
    b=order(i,2);
    if mod(a,2)==1
        coordinate(i,1)=-10;
    else
        coordinate(i,1)=10;
    end
```

```
    coordinate(i,2)=-1+4*round(a/2);
    c=mod(b,9);
    if c==0
        coordinate(i,3)=8;
    else
        coordinate(i,3)=2*(c-5);
    end
    coordinate(i,4)=2*ceil(b/9);

orderarrival=datetime(order(:,3:8));

ordertimeS(1:population_size,1:order_size)=orderarrival(1);
ordertimeC(1:population_size,1:order_size)=orderarrival(1);

AGV_num=20;

for i=1:population_size
    ordertimeS(1:population_size,1:order_size)=orderarrival(1);
    ordertimeC(1:population_size,1:order_size)=orderarrival(1);

    current=zeros(AGV_num,2);
    AGVtime(1:AGV_num,1)=orderarrival(1);
    AGVelectric(1:AGV_num,1)=order(1:AGV_num,9);
    for id=1:order_size
        distance1(population(i,id))=abs(current(population(i,id),1)-
coordinate(id,1))+abs(current(population(i,id),2)-coordinate(id,2));
        distance2(population(i,id))=abs(coordinate(id,1)-
coordinate(id,3))+abs(coordinate(id,2)-coordinate(id,4));
        distance3(population(i,id))=abs(coordinate(id,3))+abs(coordin
ate(id,4));
t1(population(i,id))=distance1(population(i,id))/population(i,id+ord
er_size);
t2(population(i,id))=distance2(population(i,id))/population(i,id+ord
er_size);
t3(population(i,id))=distance3(population(i,id))/30;
K(id,population(i,id))=0.0133*population(i,id+order_size)-0.097;
        E1(i,id)=t1(population(i,id))*K(id,population(i,id));
        E2(i,id)=t2(population(i,id))*K(id,population(i,id));
        E3(i,id)=t3((population(i,id)))*0.3;%
        unrealE(i,id)=E1(i,id)+E2(i,id)+E3(i,id);
           if AGVelectric(population(i,id))>=unrealE(i,id)
               if
(AGVtime(population(i,id))+minutes(t1(population(i,id))))<=orderarri
val(id)
                   ordertimeS(i,id)=orderarrival(id);
               else

ordertimeS(i,id)=AGVtime(population(i,id))+minutes(t1(population(i,i
d)));
               end
               realE(i,id)=E1(i,id)+E2(i,id);
               charE(i,id)=0;
           else
```

```
distance4(population(i,id))=abs(current(population(i,id),1))+abs(cur
rent(population(i,id),2));
distance5(population(i,id))=abs(coordinate(id,1))+abs(coordinate(id,
2));
t4(population(i,id))=distance2(population(i,id))/30;
t5(population(i,id))=distance5(population(i,id))/population(i,id+ord
er_size);
E4(i,id)=t4(population(i,id))*0.3;%
E5(i,id)=t3((population(i,id)))*K(id,population(i,id));
charE(i,id)=unrealE(i,id)-
AGVelectric(population(i,id))+E4(i,id)+E5(i,id)-E1(i,id);
t6(population(i,id))=(charE(i,id))/5;
            if
(AGVtime(population(i,id))+minutes(t4(population(i,id))+t5(populatio
n(i,id))+t6(population(i,id))))<=orderarrival(id)
                ordertimeS(i,id)=orderarrival(id);
            else

ordertimeS(i,id)=AGVtime(population(i,id))+minutes(t4(population(i,i
d))+t5(population(i,id))+t6(population(i,id)));
            end
            realE(i,id)=E4(i,id)+E5(i,id)+E2(i,id);
         end

ordertimeC(i,id)=ordertimeS(i,id)+minutes(t2(population(i,id)));

       current(population(i,id),:)=coordinate(id,3:4);
       AGVtime(population(i,id))=ordertimeC(i,id);

AGVelectric(population(i,id))=AGVelectric(population(i,id))+charE(i,
id)-realE(i,id);
    end

    MS(i) = max(ordertimeC(i,:));
    SMS(i) =seconds(MS(i)-orderarrival(1));
    MMS(i) =minutes(MS(i)-orderarrival(1));
    NA(i) = length(unique(population(i,1:order_size)));
    AGV(i,1:NA(i))=unique(population(i,1:order_size));
    E(i)=sum(realE(i,:));
    fitness_value(i) = 1/(0.6*SMS(i)+0.24*30*NA(i)+0.16*20*E(i));
    fitnessSMS_value(i) = SMS(i);
    fitnessNA_value(i) = NA(i) ;
    fitnessE_value(i) = E(i);
end

clear i;
clear j;
clear id;

% Rank and population entropy
function MYrank(population_size, chromosome_size)
global fitness_value;
global fitness_sum;
```

```
global fitnessSMS_value;
global fitnessNA_value;
global fitnessE_value;
global fitness_average;
global fitnessSMS_average;
global fitnessNA_average;
global fitnessE_average;
global best_fitness;
global min_fitness;
global SMS_fitness;
global NA_fitness;
global E_fitness;
global best_individual;
global best_generation;
global population;
global G;
global S;
global beta;

for i=1:population_size
    fitness_sum(i) = 0.;
end

min_index = 1;
temp = 1;
temp_chromosome(chromosome_size)=0;
for i=1:population_size
    min_index = i;
    for j = i+1:population_size
        if fitness_value(j) < fitness_value(min_index);
            min_index = j;
        end
    end

    if min_index ~= i
        temp = fitness_value(i);
        fitness_value(i) = fitness_value(min_index);
        fitness_value(min_index) = temp;
        tempSMS = fitnessSMS_value(i);
        fitnessSMS_value(i) = fitnessSMS_value(min_index);
        fitnessSMS_value(min_index) = tempSMS;
        tempNA = fitnessNA_value(i);
        fitnessNA_value(i) = fitnessNA_value(min_index);
        fitnessNA_value(min_index) = tempNA;
        tempE = fitnessE_value(i);
        fitnessE_value(i) = fitnessE_value(min_index);
        fitnessE_value(min_index) = tempE;
        for k = 1:chromosome_size
            temp_chromosome(k) = population(i,k);
            population(i,k) = population(min_index,k);
            population(min_index,k) = temp_chromosome(k);
        end
    end
end
```

```
fitnessSMS_sum(i)=0;
fitnessNA_sum(i)=0;
fitnessE_sum(i)=0;
for i=1:population_size
    if i==1
        fitness_sum(i) = fitness_sum(i) + fitness_value(i);
        fitnessSMS_sum(i) = fitnessSMS_sum(i) + fitnessSMS_value(i);
        fitnessNA_sum(i) = fitnessNA_sum(i) + fitnessNA_value(i);
        fitnessE_sum(i) = fitnessE_sum(i) + fitnessE_value(i);
    else
        fitness_sum(i) = fitness_sum(i-1) + fitness_value(i);
        fitnessSMS_sum(i) = fitnessSMS_sum(i-1) + fitnessSMS_value(i);
        fitnessNA_sum(i) = fitnessNA_sum(i-1) + fitnessNA_value(i);
        fitnessE_sum(i) = fitnessE_sum(i-1) + fitnessE_value(i);
    end
end

fitness_sum1(i)=0;
for i=1:population_size
    if i==1
        fitness_sum1(i) = fitness_sum1(i) + 1/fitness_value(i);
    else
        fitness_sum1(i) = fitness_sum1(i-1) + 1/fitness_value(i);
    end
end
fitness_average(G) = fitness_sum1(population_size)/population_size;
fitnessSMS_average(G)                                              =
fitnessSMS_sum(population_size)/population_size;
fitnessNA_average(G) = fitnessNA_sum(population_size)/population_size;
fitnessE_average(G) = fitnessE_sum(population_size)/population_size;
if G == 1
    best_fitness(1) = fitness_value(population_size);
    min_fitness(1) = fitness_value(1);
    SMS_fitness(1) = fitnessSMS_value(population_size);
    NA_fitness(1) = fitnessNA_value(population_size);
    E_fitness(1) = fitnessE_value(population_size);
    best_generation = 1;
    for j=1:chromosome_size
        best_individual(j) = population(population_size,j);
    end
else
    if fitness_value(population_size) > best_fitness(G-1)
        best_fitness(G) = fitness_value(population_size);
        SMS_fitness(G) = fitnessSMS_value(population_size);
        NA_fitness(G) = fitnessNA_value(population_size);
        E_fitness(G) = fitnessE_value(population_size);
        best_generation = G;
        for j=1:chromosome_size
            best_individual(j) = population(population_size,j);
        end
    else
        best_fitness(G) = best_fitness(G-1);
        SMS_fitness(G) = SMS_fitness(G-1);
```

```
        NA_fitness(G) = NA_fitness(G-1);
        E_fitness(G) = E_fitness(G-1);
    end
    if fitness_value(1) < min_fitness(G-1)
        min_fitness(G) = min_fitness(1);
    else
        min_fitness(G) = min_fitness(G-1);
    end
end
     range(G)=1.08*best_fitness(G)-0.92*min_fitness(G);
     number=zeros(population_size,1);
     for i=1:population_size
         for j=1:population_size
             if       fitness_value(i)>=       (0.92*min_fitness(G)+(j-
1)*range(G)/population_size)&&fitness_value(i)<
(0.92*min_fitness(G)+j*range(G)/population_size)
                 number(j)=number(j)+1;
             end
         end
     end
     for i=1:population_size
         p(i)=number(i)/population_size;
         if p(i)~=0
             S(G)=S(G)-p(i)*log( p(i));
         end
     end
     beta(G)=S(G)/log(population_size);

clear i;
clear j;
clear k;
clear min_index;
clear temp;
clear tempSMS;
clear tempNA;
clear tempE;
clear temp1;


% Selection and Elitism
function MYselection(population_size, chromosome_size, elitism)
global population;
global population_new;
global fitness_sum;

for i=1:population_size
    r = rand * fitness_sum(population_size);
    first = 1;
    last = population_size;
    mid = round((last+first)/2);
    idx = -1;
    while (first <= last) && (idx == -1)
        if r > fitness_sum(mid)
            first = mid;
```

```
        elseif r < fitness_sum(mid)
           last = mid;
        else
           idx = mid;
           break;
        end
        mid = round((last+first)/2);
        if (last - first) == 1
           idx = last;
           break;
        end
    end
   for j=1:chromosome_size
        population_new(i,j) = population(idx,j);
   end
end
if elitism
    p = population_size-1;
else
    p = population_size;
end

for i=1:p
   for j=1:chromosome_size
       population(i,j) = population_new(i,j);
   end
end

clear i;
clear j;
clear population_new;
clear first;
clear last;
clear idx;
clear mid;


% Crossover
function MYcrossover(population_size, chromosome_size)
global population;
global beta;
global fitness_value;
global best_fitness;
global G;

cross_rate1=0.6+0.3*(1-beta(G));
for i=1:2:population_size
    cross_rate3=cross_rate1*best_fitness(G)/(2*fitness_value(i));
    cross_rate4=cross_rate1*best_fitness(G)/(2*fitness_value(i+1));
    cross_rate=max( cross_rate3,cross_rate4);
    if(rand < cross_rate)
        cross_position = round(rand * 0.5 * chromosome_size);
        if (cross_position == 0 || cross_position == 1)
```

```
            continue;
        end
        for j=cross_position:0.5*chromosome_size
            temp1 = population(i,j);
            population(i,j) = population(i+1,j);
            population(i+1,j) = temp1;
        end
        for q=cross_position+0.5*chromosome_size:chromosome_size
            temp2 = population(i,q);
            population(i,q) = population(i+1,q);
            population(i+1,q) = temp2;
        end
    end
end

clear i;
clear j;
clear q;
clear temp1;
clear temp2;
clear cross_position;


% Mutation
function MYmutation(population_size, chromosome_size)
global population;
global beta;
global fitness_value;
global best_fitness;
global G;

AGV_num=20;
mutate_rate1=0.04+0.06*(1-beta(G));
for j=1:chromosome_size
     NAX(j) = length(unique(population(:,j)));
     if j==1
        NAX_sum(j) = NAX(j);
     else
        NAX_sum(j) = NAX_sum(j-1)+NAX(j);
     end
end
for i=1:population_size
    mutate_rate=mutate_rate1*best_fitness(G)/(2*fitness_value(i));
    if rand < mutate_rate
    r = rand * NAX_sum(0.5*chromosome_size);
    first = 1;
    last = 0.5*chromosome_size;
    mid = round((last+first)/2);
    idx = -1;
    while (first <= last) && (idx == -1)
        if r > NAX_sum(mid)
            first = mid;
        elseif r < NAX_sum(mid)
```

```
            last = mid;
        else
            idx = mid;
            break;
        end
        mid = round((last+first)/2);
        if (last - first) == 1
            idx = last;
            break;
        end
    end
        mutate_position = idx;
        temp1=population(i,mutate_position);
        for j=1:100
            temp2 = randperm(AGV_num,1);
            if temp2~=temp1
                population(i,mutate_position) = temp2;
                break;
            end
        end
    end
    if rand < mutate_rate
    r = rand * (NAX_sum(chromosome_size)-NAX_sum(0.5*chromosome_size));
    last = chromosome_size;
    mid = round((last+first)/2);
    idx = -1;
    while (first <= last) && (idx == -1)
        if r > NAX_sum(mid)
            first = mid;
        elseif r < NAX_sum(mid)
            last = mid;
        else
            idx = mid;
            break;
        end
        mid = round((last+first)/2);
        if (last - first) == 1
            idx = last;
            break;
        end
    end
        mutate_position =idx;
        temp3=population(i,mutate_position);
        for q=1:100
            temp4=60*(0.4+0.1*randperm(11,1));
            if temp4~=temp3
                population(i,mutate_position) = temp4;
                break;
            end
        end
    end
end

clear i;
```

```
clear j;
clear q;
clear temp1;
clear temp2;
clear temp3;
clear temp4;
clear mutate_position;


% Algorithmic representation and population entropy mapping
function MYplotGA(generation_size)
global fitness_average;
global fitnessSMS_average;
global fitnessNA_average;
global fitnessE_average;
global best_fitness;
global SMS_fitness;
global NA_fitness;
global E_fitness;
global S;
x = 1:1:generation_size;
y1 = fitness_average(x);
y2 = 1./best_fitness;
y3 = fitnessSMS_average(x);
y4 = SMS_fitness;
y5 = fitnessNA_average(x);
y6 = NA_fitness;
y7 = fitnessE_average(x);
y8 = E_fitness;
y9 = S;

figure(1);
subplot(2,1,1)
plot(x,y1)
subplot(2,1,2)
plot(x,y2)
figure(2);
subplot(4,2,1)
plot(x,y1)
subplot(4,2,2)
plot(x,y2)
subplot(4,2,3)
plot(x,y3)
subplot(4,2,4)
plot(x,y4)
subplot(4,2,5)
plot(x,y5)
subplot(4,2,6)
plot(x,y6)
subplot(4,2,7)
plot(x,y7)
subplot(4,2,8)
plot(x,y8)
figure(3);
```

```
plot(x,y9)



% the Gantt chart
function MYorder_job()
global best_individual;
figure(4);
population = best_individual;
population_size = 100;
chromosome_size = 100;
    fitness_value= 0;
    fitnessSMS_value = 0;
    fitnessNA_value = 0;
    fitnessE_value = 0;
    distance1=0;
    distance2=0;
    distance3=0;
    distance4=0;
    distance5=0;
    distance6=0;
    t1=0;
    t2=0;
    t3=0;
    t4=0;
    t5=0;
    t6=0;
order=xlsread('order data.xlsx');
order_size=0.5*chromosome_size;
coordinate=zeros(order_size,4);
for i=1:order_size
    a=order(i,1);
    b=order(i,2);
    if mod(a,2)==1
        coordinate(i,1)=-10;
    else
        coordinate(i,1)=10;
    end
    coordinate(i,2)=-1+4*round(a/2);
    c=mod(b,9);
    if c==0
        coordinate(i,3)=8;
    else
        coordinate(i,3)=2*(c-5);
    end
    coordinate(i,4)=2*ceil(b/9);
end
orderarrival=datetime(order(:,3:8));
ordertimeS(1,1:order_size)=orderarrival(1);
ordertimeC(1,1:order_size)=orderarrival(1);
AGV_num=20;
    for i=1
    ordertimeS(1,1:order_size)=orderarrival(1);
    ordertimeC(1,1:order_size)=orderarrival(1);
```

```
    char_timeS(1,1:order_size)=orderarrival(1);
    char_timeC(1,1:order_size)=orderarrival(1);
    wating_timeS(1,1:order_size)=orderarrival(1);
    wating_timeC(1,1:order_size)=orderarrival(1);
    orderjobtimeS(1,1:order_size)=orderarrival(1);
    orderjobtimeC(1,1:order_size)=orderarrival(1);
    current=zeros(AGV_num,2);
    AGVtime(1:AGV_num,1)=orderarrival(1);
    AGVelectric(1:AGV_num,1)=order(1:AGV_num,9);
    for id=1:order_size

        distance1(population(i,id))=abs(current(population(i,id),1)-
coordinate(id,1))+abs(current(population(i,id),2)-coordinate(id,2));
        distance2(population(i,id))=abs(coordinate(id,1)-
coordinate(id,3))+abs(coordinate(id,2)-coordinate(id,4));

distance3(population(i,id))=abs(coordinate(id,3))+abs(coordinate(id,
4));
t1(population(i,id))=distance1(population(i,id))/population(i,id+ord
er_size);
t2(population(i,id))=distance2(population(i,id))/population(i,id+ord
er_size);
        t3(population(i,id))=distance3(population(i,id))/30;
        K(id,population(i,id))=0.0133*population(i,id+order_size)-
0.097;
        E1(i,id)=t1(population(i,id))*K(id,population(i,id));
        E2(i,id)=t2(population(i,id))*K(id,population(i,id));
        E3(i,id)=t3((population(i,id)))*0.3;
        unrealE(i,id)=E1(i,id)+E2(i,id)+E3(i,id);
           if AGVelectric(population(i,id))>=unrealE(i,id)
               orderjobtimeS(id)=AGVtime(population(i,id));

orderjobtimeC(id)=AGVtime(population(i,id))+minutes(t1(population(i,
id)));
               if orderjobtimeC(id)<=orderarrival(id)
                   ordertimeS(i,id)=orderarrival(id);
                   wating_timeS(id)=orderjobtimeC(id);
                   wating_timeC(id)=ordertimeS(i,id);
               else
                   ordertimeS(i,id)=orderjobtimeC(id);
               end
               realE(i,id)=E1(i,id)+E2(i,id);
               charE(i,id)=0;
           else

distance4(population(i,id))=abs(current(population(i,id),1))+abs(cur
rent(population(i,id),2));
               t4(population(i,id))=distance2(population(i,id))/30;
distance5(population(i,id))=abs(coordinate(id,1))+abs(coordinate(id,
2));

t5(population(i,id))=distance5(population(i,id))/population(i,id+ord
er_size);
               E4(i,id)=t4(population(i,id))*0.3;
```

```
            E5(i,id)=t3((population(i,id)))*K(id,population(i,id));
            charE(i,id)=unrealE(i,id)-
AGVelectric(population(i,id))+E4(i,id)+E5(i,id)-E1(i,id);
            t6(population(i,id))=(charE(i,id))/5;
orderjobtimeS(id)=AGVtime(population(i,id))+minutes(t4(population(i,
id))+t6(population(i,id)));
            char_timeS(id)=AGVtime(population(i,id));

char_timeC(id)=AGVtime(population(i,id))+minutes(t4(population(i,id)
)+t6(population(i,id)));
            orderjobtimeS(id)=char_timeC(id);

orderjobtimeC(id)=char_timeC(id)+minutes(t5(population(i,id)));
            if orderjobtimeC(id)<=orderarrival(id)
               ordertimeS(i,id)=orderarrival(id);
               wating_timeS(id)=orderjobtimeC(id);
               wating_timeC(id)=ordertimeS(i,id);
            else
               ordertimeS(i,id)=orderjobtimeC(id);
            end
            realE(i,id)=E4(i,id)+E5(i,id)+E2(i,id);
         end
ordertimeC(i,id)=ordertimeS(i,id)+minutes(t2(population(i,id)));

      current(population(i,id),:)=coordinate(id,3:4);
      AGVtime(population(i,id))=ordertimeC(i,id);
AGVelectric(population(i,id))=AGVelectric(population(i,id))+charE(i,
id)-realE(i,id);
     end
   end
axis([0,200,0,20.5]);
set(gca,'xtick',0:10:200) ;
set(gca,'ytick',0:1:10.5) ;
xlabel('Time'),ylabel('AGV ID');
title('Task');
n_bay_nb=20;
n_task_nb = 4*order_size;
for i=1:order_size
n_start_time(i)=seconds(ordertimeS(i)-orderarrival(1));
n_start_time(i+order_size)=seconds(char_timeS(i)-orderarrival(1));
n_start_time(i+2*order_size)=seconds(wating_timeS(i)-
orderarrival(1));
n_start_time(i+3*order_size)=seconds(orderjobtimeS(i)-
orderarrival(1));
end
for i=1:order_size
n_duration_time(i)=seconds(ordertimeC(i)-ordertimeS(i));
n_duration_time(i+order_size)=seconds(char_timeC(i)-char_timeS(i));
n_duration_time(i+2*order_size)=seconds(wating_timeC(i)-
wating_timeS(i));
n_duration_time(i+3*order_size)=seconds(orderjobtimeC(i)-
orderjobtimeS(i));
end
for i=1:order_size
```

```
n_bay_start(i)=population(i)-1;
n_bay_start(i+order_size)=population(i)-1;
n_bay_start(i+2*order_size)=population(i)-1;
n_bay_start(i+3*order_size)=population(i)-1;
end
for i=1:order_size
n_job_id(i)=0;
n_job_id(i+order_size)=1;
n_job_id(i+2*order_size)=2;
n_job_id(i+3*order_size)=3;
end
rec=[0,0,0,0];
color=['c','g','m','y'];
for i =1:n_task_nb
  rec(1) = n_start_time(i);
  rec(2) = n_bay_start(i)+0.7;
  rec(3) = n_duration_time(i);
  rec(4) = 0.6;
  j=mod(i,order_size);
  if j==0
      txt=sprintf('%d-%2.1f',order_size,population(2*order_size)/60);
  else
      txt=sprintf('%d-%2.1f',j,population(j+order_size)/60);
  end
   rectangle('Position',rec,'LineWidth',0.5,'LineStyle','-
','FaceColor',color(n_job_id(i)+1));
   if n_duration_time(i)>0
       if i<= order_size
          if j==0
              txt=sprintf('%d-%2.1f',order_size,population(2*order_siz
e)/60);
           else
              txt=sprintf('%d-%2.1f',j,population(j+order_size)/60);
          end
       end
       if i> 3*order_size
           if j==0

txt=sprintf('%d-%2.1f',order_size,population(2*order_size)/60);
str=sprintf('%d:%2.1f',order_size,realE(order_size));
text(n_start_time(i)+0.2,(n_bay_start(i)+1.5),str,'FontWeight','Bold
','FontSize',10);
           else
              txt=sprintf('%d-%2.1f',j,population(j+order_size)/60);
              str=sprintf('%d:%2.1f',j,realE(j));

text(n_start_time(i)+0.2,(n_bay_start(i)+1.5),str,'FontWeight','Bold
','FontSize',10);
           end
       end
       if i> order_size && i<= 3*order_size
           if j==0
              txt=sprintf('%d',order_size);
            else
```

```
            txt=sprintf('%d',j);
         end
      end
   end
text(n_start_time(i)+0.2,(n_bay_start(i)+1),txt,'FontWeight','Bold',
'FontSize',10);
end
clear;


% the main of MAGA
function      [m,n,p]      =      MYgenetic_algorithm(population_size,
chromosome_size, generation_size, elitism)
global G ;
global fitness_value;
global best_fitness;
global SMS_fitness;
global NA_fitness;
global E_fitness;
global fitness_average;
global fitnessSMS_average;
global fitnessNA_average;
global fitnessE_average;
global S;
global best_individual;
global best_generation;

fitness_average = zeros(generation_size,1);
fitnessSMS_average = zeros(generation_size,1);
fitnessNA_average = zeros(generation_size,1);
fitnessE_average = zeros(generation_size,1);
best_fitness = zeros(generation_size,1);
SMS_fitness = zeros(generation_size,1);
NA_fitness = zeros(generation_size,1);
E_fitness = zeros(generation_size,1);
S = zeros(generation_size,1);


fitness_value(population_size) = 0;
best_generation = 0;

MYinit(population_size, chromosome_size);

for G=1:generation_size
   MYfitness(population_size, chromosome_size);
   MYrank(population_size, chromosome_size);
   MYselection(population_size, chromosome_size, elitism);
   MYcrossover(population_size, chromosome_size);
   MYmutation(population_size, chromosome_size);
end

MYplotGA(generation_size);
MYorder_job();
```

```
m = best_individual;
n = 1/best_fitness(G);

clear i;
clear j;


% to run
function MYtest()
t0=cputime;
elitism = true;
population_size = 100;
chromosome_size = 100;
generation_size = 500;

[best_individual,best_fitness1,iterations]=MYgenetic_algorithm(popul
ation_size, chromosome_size, generation_size,elitism);

disp best_individual:
best_individual
disp f(x):
best_fitness1
disp CPU time:
t=cputime-t0;
t
clear;
```
