# Supplementary material for: Multi-objective AGV scheduling in an automatic sorting system of an unmanned (intelligent) warehouse by using two adaptive genetic algorithms and a multi-adaptive genetic algorithm
Source: PLoS One. 2019 Dec 6;14(12):e0226161. doi: 10.1371/journal.pone.0226161 (PMC6897425; doi:10.1371/journal.pone.0226161)
Supplement: Attachment — Submitted filename: Response to Reviewers.docx [file pone.0226161.s003.docx]

Dear Editor Wang and Reviewers:

We wish to thank you for the time and effort you have spent reviewing our paper ‘Multi-objective AGV scheduling in an automatic sorting system of an unmanned (intelligent) warehouse by using two adaptive genetic algorithms and a multi-adaptive genetic algorithm’ (ID: PONE-D-19-14670). We are pleased to note that you have found our research work interesting and also pointed out some problems to help us improve the quality of our work. Thank you very much for giving us an opportunity to revise our manuscript.

Our itemized response to your questions, comments and suggestions is as follows:

**Journal Requirements**

1. When submitting your revision, we need you to address these additional requirements.

Please ensure that your manuscript meets PLOS ONE's style requirements, including those for file naming. The PLOS ONE style templates can be found at

<http://www.journals.plos.org/plosone/s/file?id=wjVg/PLOSOne_formatting_sample_main_body.pdf> and <http://www.journals.plos.org/plosone/s/file?id=ba62/PLOSOne_formatting_sample_title_authors_affiliations.pdf>

**Response ：**

**Our manuscript and file naming meet the requirements of PLOS ONE.**

2. We suggest you thoroughly copyedit your manuscript for language usage, spelling, and grammar. If you do not know anyone who can help you do this, you may wish to consider employing a professional scientific editing service.

Upon resubmission, please provide the following:

- The name of the colleague or the details of the professional service that edited your manuscript
- A copy of your manuscript showing your changes by either highlighting them or using track changes (uploaded as a *supporting information* file)
- A clean copy of the edited manuscript (uploaded as the new *manuscript* file)

**Response ：**

**We have asked some English teachers and native English-language speakers to help us revise the manuscript. The revised manuscripts have been uploaded as required. We highlighted the changes with red fonts and underlines in 'Revised Manuscript with Track Changes’.**

3. Please amend either the title on the online submission form (via Edit Submission) or the title in the manuscript so that they are identical.

**Response ：**

**We are very sorry for our negligence, and we have revised the title of the manuscript.**

4. Thank you for stating the following in the Competing Interests section: "The authors have declared that no competing interests exist."

We note that one or more of the authors are employed by a commercial company:

"Beijing capital international airport Company Limited".

a) Please provide an amended Funding Statement declaring this commercial affiliation, as well as a statement regarding the Role of Funders in your study. If the funding organization did not play a role in the study design, data collection and analysis, decision to publish, or preparation of the manuscript and only provided financial support in the form of authors' salaries and/or research materials, please review your statements relating to the author contributions, and ensure you have specifically and accurately indicated the role(s) that these authors had in your study. You can update author roles in the Author Contributions section of the online submission form.

Please also include the following statement within your amended Funding Statement.

“The funder provided support in the form of salaries for authors [insert relevant initials], but did not have any additional role in the study design, data collection and analysis, decision to publish, or preparation of the manuscript. The specific roles of these authors are articulated in the ‘author contributions’ section.”

If your commercial affiliation did play a role in your study, please state and explain this role within your updated Funding Statement.

b)  Please also provide an updated Competing Interests Statement declaring this commercial affiliation along with any other relevant declarations relating to employment, consultancy, patents, products in development, or marketed products, etc.

Within your Competing Interests Statement, please confirm that this commercial affiliation does not alter your adherence to all PLOS ONE policies on sharing data and materials by including the following statement: "This does not alter our adherence to  PLOS ONE policies on sharing data and materials.” (as detailed online in our guide forauthors

<http://journals.plos.org/plosone/s/competing-interests>) . If this adherence statement is not accurate and  there are restrictions on sharing of data and/or materials, please state these. Please note that we cannot proceed with consideration of your article until this information has been declared.) . If this adherence statement is not accurate and  there are restrictions on sharing of data and/or materials, please state these. Please note that we cannot proceed with consideration of your article until this information has been declared.

Please include both an updated Funding Statement and Competing Interests Statement in your cover letter. We will change the online submission form on your behalf.

Please know it is PLOS ONE policy for corresponding authors to declare, on behalf of all authors, all potential competing interests for the purposes of transparency. PLOS defines a competing interest as anything that interferes with, or could reasonably be perceived as interfering with, the full and objective presentation, peer review, editorial decision-making, or publication of research or non-research articles submitted to one of the journals. Competing interests can be financial or non-financial, professional, or personal. Competing interests can arise in relationship to an organization or another person. Please follow this link to our website for more details on competing interests:

<http://journals.plos.org/plosone/s/competing-interests>

**Response ：**

a) **Zengrong Su contributes to our paper as an individual, and her contributions have been mentioned in the Author Contributions.**

**Funding Statement: This research was supported by the National Key R&D Program of China (No. 2018YFB1601603). The financial support of this research has nothing to do with ‘Beijing capital international airport Company Limited’.**

b) **Competing Interests Statement: Our paper has no commercial connection with Beijing capital international airport Company Limited, so this does not alter our adherence to PLOS ONE policies on sharing data and materials.**

**Replies to the reviewers’ comments**

Reviewer #1: Authors may revise this paper according to suggestions below:
1. The major contributions of this paper are not clear. Author should specifically describe them.

**Response:**

**We have described the contribution of this paper in the conclusion section before, but it may not be prominent enough. We have rewritten the content in separate paragraphs in revised manuscript (line 473 – line 481).**

**The major contributions of this paper are as follows:**

- **The work discussed in this paper can effectively improve the operation efficiency of AGV in enterprise intelligent warehouses.**
- **The multi-adaptive genetic algorithm proposed in this paper has strong global search ability and fast optimization speed, which may help other scholars get the approximate optimal solution faster and better under the same conditions.**
- **The scheduling model developed in this paper may inspire AGV scheduling in different research fields, such as FMS, to take speed as one of the variables and energy consumption as one of the objectives.**

2. Authors may polish writing of this paper.

**Response:**

**We have polished writing of this paper with the help of English teachers and our native English speaking friends.**

Reviewer #2: The research is interesting and meaningful. The makespan, the number of AGVs, and electricity consumption were taken into consideration in the scheduling process for unmanned warehouse. A multi-adaptive genetic algorithm was proposed to solve the model. The structure is clear. However, before recommending it for publication, there still need several clarifications that are listed below.
(1) In the part of Model derivation, some expressions of formulas are not standard, particularly the objective function. And equation (1) is quite confusing. I suggest explaining the equations (1)-(10), as well as other unexplained formulas, to allow a better understanding experience.

**Response:**

**We have rewritten this part according to the reviewer’s suggestion**

**We have modified the expression of makespan, adjusted the order of the equations (1)-(10), and added explanations of them in revised manuscript (line 193 – line 215).**

(2) Is there any criteria for choosing the weights and coefficients of the three objectives?

**Response:**

**The weights are determined by the degree of attention paid to the three objectives in the actual intelligent warehouse and the core idea of Pareto optimality.**

**The coefficients are used to adjust the values of the three objectives to have similar ranges of variation, so they are determined based on the range of variation of the three objectives.**

(3) There are lots of mistakes in the paper (e.g., some data in Table 4 is incorrect; Fig. 3 is in consistent with the description; the title of figures are not standardized), please check the whole carefully.

**Response:**

**Thank you for pointing them out. We are very sorry for our incorrect writing, and we have revised them.**

(4) Please put your contributions clear in the part of conclusion.

**Response:**

**We have rewritten our contributions in separate paragraphs in the part of conclusion (line 473 – line 481).**

**The major contributions of this paper are as follows:**

- **The work discussed in this paper can effectively improve the operation efficiency of AGV in enterprise intelligent warehouses.**
- **The multi-adaptive genetic algorithm proposed in this paper has strong global search ability and fast optimization speed, which may help other scholars get the approximate optimal solution faster and better under the same conditions.**
- **The scheduling model developed in this paper may inspire AGV scheduling in different research fields, such as FMS, to take speed as one of the variables and energy consumption as one of the objectives.**

(5) The language should be improved.

**Response:**

**We have polished writing of this paper with the help of English teachers and our native English speaking friends.**

(6) Please cite the following references:

Wang et al., 2017. Profit Allocation in Collaborative Multiple Centers Vehicle Routing Problem. Journal of Cleaner Production, 144:203-219.

**Response:**

**We read the article carefully and found it very helpful to us - some of our revisions are based on the inspiration of this article. And we have cited it in revised manuscript(line 99 – line 101).**

Once again, thank you very much for your constructive comments and suggestions which would help us both in English and in depth to improve the quality of the paper.

Kind regards,

Yubang Liu

XXX

E-mail: 17120842@bjtu.edu.cn

Corresponding author : Shouwen JI; Yubang Liu

E-mail: [swji@bjtu.edu.cn](mailto:swji@bjtu.edu.cn); 17120842@bjtu.edu.cn
