# Supplementary material for: Multi-objective AGV scheduling in an automatic sorting system of an unmanned (intelligent) warehouse by using two adaptive genetic algorithms and a multi-adaptive genetic algorithm
Source: PLoS One. 2019 Dec 6;14(12):e0226161. doi: 10.1371/journal.pone.0226161 (PMC6897425; doi:10.1371/journal.pone.0226161)
Supplement: Attachment — Submitted filename: Response to Reviewers.docx [file pone.0226161.s004.docx]

Dear Editor Wang and Reviewers:

We wish to thank you for the time and effort you have spent reviewing our paper ‘Multi-objective AGV scheduling in an automatic sorting system of an unmanned (intelligent) warehouse by using two adaptive genetic algorithms and a multi-adaptive genetic algorithm’ (ID: PONE-D-19-14670). We are pleased to note that you have found our research work interesting and also pointed out some problems to help us improve the quality of our work. Thank you very much for giving us the opportunity to modify the manuscript again.

Our itemized response to your questions, comments and suggestions is as follows:

**Replies to the reviewers’ comments**

Reviewer #2: I have no further comments. The authors have answered all my questions. This paper can be accepted.

**Response:**

**We appreciate your approval of our paper. Thank you for your suggestions and guidance on our article before.**

Reviewer #3: The manuscript addresses an optimal loading model for AGV scheduling in an automatic sorting system by using improved adaptive genetic algorithms. The topic of this paper is interesting. However, this paper has a number of major shortcomings that need to be addressed:

(1) The contributions of this paper to the literature should be clear.

**Response:**

**We have rewritten our contributions in a separate paragraph in the part of conclusion (line 473 – line 481).**

**The major contributions of this paper are as follows:**

- **The work discussed in this paper can effectively improve the operation efficiency of AGV in enterprise intelligent warehouses.**
- **The multi-adaptive genetic algorithm proposed in this paper has strong global search ability and fast optimization speed, which may help other scholars get the approximate optimal solution faster and better under the same conditions.**
- **The scheduling model developed in this paper may inspire AGV scheduling in different research fields, such as FMS, to take speed as one of the variables and energy consumption as one of the objectives.**

(2) Authors are kindly encouraged to add some latest literature on the heuristic algorithms, optimization model of scheduling problem and vehicle routing problem.

Zhang, D., Wang, X., Li, S., Ni, N., Zhang, Z., 2018a. Joint optimization of green vehicle scheduling and routing problem with time-varying speeds. PLoS One, 1-13.

Zhang, D.Z., Zou, F.Z., Li, S.Y., Zhou, L.Y., 2017. Green Supply Chain Network Design with Economies of Scale and Environmental Concerns. J. Adv. Transp., 1-14.

Yong Wang, Xiaolei Ma, Maozeng Xu, Yinhai Wang, Yong Liu. 2015. Vehicle Routing Problem based on A Fuzzy Customer Clustering Approach for Logistics Network Optimization, Journal of Intelligent&Fuzzy Systems, 29: 1427-1442.

**Response:**

**We read these articles carefully and we have cited them in revised manuscript(line 92 – line 94).**

(3) In the section of “Algorithm design”, authors are kindly encouraged to describe clear on the repairing the infeasible solution.

**Response:**

**We would like to explain: each solution in this paper consists of two halves, the first half is the allocation of the order sorting work, and the second half is the speed of the AGV when the order is sorted.**

**If a random solution is an infeasible solution, then there should be one of two situations: battery limited or time conflict. However, the charging task is considered in the mathematical model of this paper. As long as the power of the AGV is insufficient, the charging task will be triggered: the charging time will be no longer 0, it will be calculated (line220). So, there will be no limited of battery.**

**In addition, the various times (**$\boldsymbol{t}\mathbf{ST}_{\boldsymbol{i}}$**,**$\boldsymbol{t}\mathbf{CT}_{\boldsymbol{i}}$**,…) in this paper are calculated by the composition of the solution (line 202-207, line 216-221), so the task time of different orders sorted by the same AGV does not conflict.**

**So, even if it is extreme to assign all orders to the same AGV to complete the order picking, it is still a feasible solution with the longer charging time. Therefore, the solutions generated under the rules set in this paper are all feasible solutions, and there are no infeasible solutions.**

(4) In the section of “Computational results and discussion “, authors are kindly encouraged to give some comparative analysis on the two proposed algorithms (i.e. AGA and MAGA)with other optimization solver ( e.g. Cplex ) on the small-size instances.

**Response:**

**Thank you for pointing it out. In fact, at the earliest time, we really thought about doing such a comparison. Our idea at the time was to use LINGO to compare with our proposed MAGA. But after the experiment, we found that the effect of using LINGO is very unsatisfactory. Because the research in this paper not only involves the assignment of order sorting, but also the speed is variable, even if the solution space of the small-scale example reaches 1.75*10^61, it is very difficult to solve with LINGO. So in the end, we chose to use our proposed MAGA to compare with the traditional AGA proposed by others.** **Therefore, we hope that you understand that there is no good effect in using CPLEX. We think that our algorithm (MAGA) is better than AGA, and this is enough to reflect the superior performance of the MAGA.**

Once again, thank you very much for your constructive comments and suggestions which would help us both in English and in depth to improve the quality of the paper.

Kind regards,

Yubang Liu

E-mail: 17120842@bjtu.edu.cn

Corresponding author : Shouwen JI; Yubang Liu

E-mail: [swji@bjtu.edu.cn](mailto:swji@bjtu.edu.cn); 17120842@bjtu.edu.cn
